# Corrigendum: Argon treatment after experimental subarachnoid hemorrhage: evaluation of microglial activation and neuronal survival as a subanalysis of a randomized controlled animal trial

**DOI:** 10.4103/mgr.MEDGASRES-D-25-00322

**Published:** 2026-01-06

In the article titled “Argon treatment after experimental subarachnoid hemorrhage: evaluation of microglial activation and neuronal survival as a subanalysis of a randomized controlled animal trial,” published on pages 103–109, Issue 3, Volume 10, 2020 of *Medical Gas Research*^1^ (doi: 10.4103/2045-9912.296039), the labels for SAH Ar and SAH N_2_ groups in **Figures 1, 4–6** were incorrectly written as “Sham Ar” and “Sham N_2_” in the published version. The correct **Figures 1**, **4–6** are shown below:


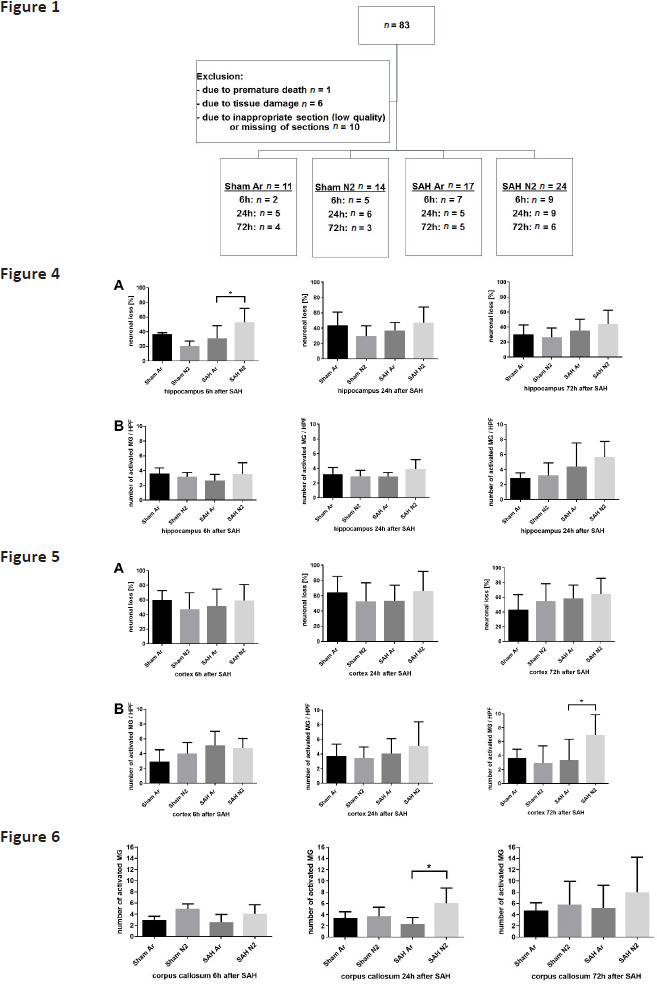


Additionally, **Figure 3A** shows an exemplary brain slice including the regions of interest analyzed. This figure is not a part of the analyzed data but merely an internal laboratory template for precisely defining anatomical locations, ultimately serving quality assurance. Therefore, the title of **Figure 3A** is corrected as exemplary representation of the anatomical locations analyzed (modified from Veldeman et al.^2^).

This correction does not change the results, interpretations, or conclusions of the study. The authors apologize for any inconvenience this correction may cause for readers and editors of *Medical Gas Research*.
